# Metabolic signaling in T cells

**DOI:** 10.1038/s41422-020-0379-5

**Published:** 2020-07-24

**Authors:** Justin A. Shyer, Richard A. Flavell, Will Bailis

**Affiliations:** 1 Department of Immunobiology, Yale School of Medicine, New Haven, CT USA; 2 Howard Hughes Medical Institute, Chevy Chase, MD USA; 3 Pathology and Laboratory Medicine, Children’s Hospital of Philadelphia, Philadelphia, PA USA

**Keywords:** Immunology, Molecular biology

## Abstract

The maintenance of organismal homeostasis requires partitioning and transport of biochemical molecules between organ systems, their composite cells, and subcellular organelles. Although transcriptional programming undeniably defines the functional state of cells and tissues, underlying biochemical networks are intricately intertwined with transcriptional, translational, and post-translational regulation. Studies of the metabolic regulation of immunity have elegantly illustrated this phenomenon. The cells of the immune system interface with a diverse set of environmental conditions. Circulating immune cells perfuse peripheral organs in the blood and lymph, patrolling for pathogen invasion. Resident immune cells remain in tissues and play more newly appreciated roles in tissue homeostasis and immunity. Each of these cell populations interacts with unique and dynamic tissue environments, which vary greatly in biochemical composition. Furthermore, the effector response of immune cells to a diverse set of activating cues requires unique cellular adaptations to supply the requisite biochemical landscape. In this review, we examine the role of spatial partitioning of metabolic processes in immune function. We focus on studies of lymphocyte metabolism, with reference to the greater immunometabolism literature when appropriate to illustrate this concept.

## Introduction

The immune system is the critical effector of host defense from pathogens. Beyond this role, immune cells are important regulators of wound repair, tissue remodeling, and basal tissue function. Immunodeficiencies result in susceptibility to infection, increased incidence of cancer, and cognitive and developmental defects. On the other end of the spectrum, hyperactivation of the immune system results in autoimmune and autoinflammatory disorders including diabetes, inflammatory bowel disease, and systemic lupus erythematosus. To achieve such a broad range of function, the immune system has evolved to be highly adaptive. Individual immune cells are capable of adopting multiple functional programs in response to specific sets of stimuli. The functional programs of immune cells differ in their differentiation signals, transcriptional regulators, and effector molecules. However, a more recent body of literature has described a now well appreciated role for cellular metabolism in regulating immune cell plasticity. Adaptations in cellular metabolism that accompany transcriptional reprogramming are required for immune cells to meet the biochemical demands of each distinct functional state. Intriguingly, the remodeling of biochemical networks also acts upstream of signal transduction and chromatin remodeling, suggesting that cellular metabolism acts as more than subservient transcriptional nodes downstream of receptor signaling.

## Programming of T cell metabolism

Distinct T cell activation states require metabolic programs compatible with their functional demands. The transition between states is accompanied by active reprogramming of cellular metabolism. Naïve T cells rapidly rewire metabolic networks upon activation to meet the demands of clonal expansion and epigenetic remodeling. Activated effector T cells adopt one of a myriad of functional programs, each with distinct biochemical demands. Long-lived memory T cells exhibit a quiescent program but maintain a primed state. Compared to naïve T cells, memory cells more rapidly take up glucose, engage in glycolysis, and more efficiently utilize glucose for fatty acid synthesis upon antigen rechallenge.^1–3^ Each transition requires coordinated adaptations orchestrated by a series of signal transduction and transcription factor networks. In this section, we review the mechanisms that govern the programming of metabolism in activated T cells.

### Exiting the naïve state

**Fig. 1:**
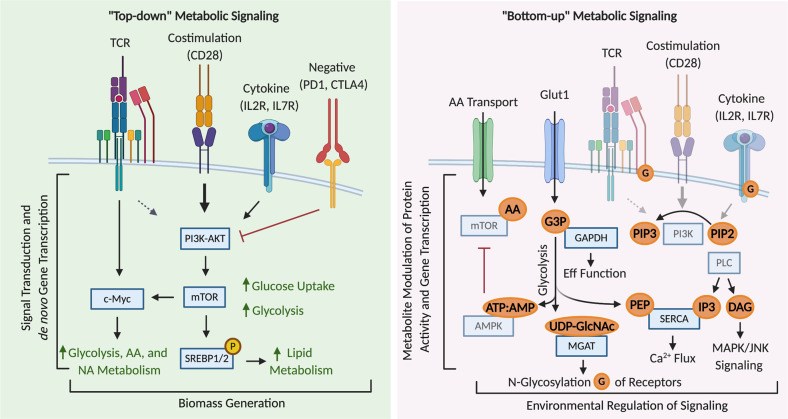
“Top-down” vs “bottom-up” metabolic signaling during T cell activation. “Top-down” signaling regulates the programming of T cell metabolism downstream of ligation of the TCR, co-stimulation and cytokine signaling. Key metabolic regulators are engaged to meet the bioenergetic demands of effector T cells. Signal transduction pathways and de novo gene transcription lead to increased transcription and activation of mTOR and c-Myc, two master regulators of anabolism. mTOR and c-Myc are required to increase glucose uptake and metabolism. c-Myc is also critical for increasing amino acid (AA) and nucleic acid (NA) metabolism. mTOR activates increased lipid metabolism through SREBP1/2. “Bottom-up” signaling refers to metabolite regulation of signaling effectors. Increased rates of glucose and amino acid uptake and metabolism lead to the generation of metabolites that modulate the activity of several key signaling effectors, a process termed bottom-up metabolic signaling. Levels of glycolytic intermediates alter the activity of RNA-binding proteins, regulate post-translational glycosylation, and activate the key metabolic regulator AMPK. Amino acid metabolism and uptake regulate mTOR activity though multiple mechanisms. Lipid species regulate the activity of several key signaling effectors of T cell activation.

T cell activation is initiated by stimulation of the T cell receptor (TCR) complex through engagement of cognate peptide-MHC complexes as well as the ligation of the co-receptor CD28 by co-stimulatory molecules on an antigen presenting cell.^4–6^ TCR activation and co-stimulation initiate discrete sets of signaling cascades that collectively license a T cell to exit quiescence. Broadly, stimulation of the TCR promotes signaling through the ERK/MAPK pathways and calcium flux, CD28 signaling activates the PI3K-AKT-mTOR axis, and both pathways together engage the NF-κB pathway.^7,8^ In addition, growth factors, such as IL-2, can stimulate PI3K-AKT-mTOR signaling and the TCR has also been implicated in activating the pathway.^9–14^ Of these signal transduction pathways, the PI3K-AKT-mTOR axis and Myc signaling are understood to be the primary regulators of early metabolic changes associated with T cell activation and differentiation (Fig. 1).^15,16^

#### PI3K signaling and T cell metabolism

The primary metabolic adaptation of an activating T cell is an increase in glucose metabolism. Co-stimulation signaling downstream of CD28 activation licenses glucose uptake from the extracellular environment. Although TCR stimulation is necessary, CD28-mediated signaling is the primary regulator of expression of the plasma membrane glucose transporter, Glut1.^17^ In activated T cells as in other cell types, the PI3K-AKT signaling axis is required for increases in Glut1 expression and glucose uptake.^17–19^ Although increasing the strength of stimulation through the TCR can license T cell glucose import in the absence of co-stimulation, the magnitude of glucose import in this setting is markedly diminished.^19^ Glut1 expression is required for functional effector T cell responses.^20^ AKT-mediated regulation of Glut1 expression and glucose uptake is not unique to T cells activating from the naïve state. Homeostatic maintenance of T cells by IL-7 also requires PI3K-AKT signaling to sustain Glut1 expression, through a mechanism dependent on STAT5 signaling.^21,22^ Memory cells remain dependent on CD28-mediated PI3K-AKT signaling to induce Glut1 expression upon reactivation.^2^ However, memory T cells employ additional mechanisms, including Notch signaling, to enhance AKT activation, Glut1 expression, and glucose uptake.^23^

In contrast to effector T cells, the extent to which regulatory T cells (Tregs) engage PI3K-AKT signaling to promote glucose utilization and the role of glycolysis in Treg biology remain less clear. Foxp3 has been reported to suppress PI3K-AKT-mediated Glut1 upregulation and the glycolytic program through to maintain suppressive capacity and survival in low glucose environments.^24,25^ In contrast to murine Tregs, human Tregs must maintain elevated glycolytic activity to retain optimal suppressor function.^26^ Moreover, other groups have found that unlike peripheral Tregs, thymic Tregs upregulate Glut1 expression to levels comparable to T helper type 1 (Th1) cells and display similar rates of glycolysis.^27^ Given that exposure to TGF-β results in diminished glycolysis and PI3K signaling in both peripheral and thymic Tregs, it is likely that exposure to this important cytokine explains some of these reported differences in Treg metabolism. Further work is needed to better deconvolute the relationship of the PI3K-AKT pathway and TGF-β signaling in regulating peripheral versus thymic Treg metabolism.

Pathways that antagonize co-stimulation, including signaling downstream of the co-inhibitory receptors CTLA4 and PD-1, impair glucose uptake and metabolism. Engagement of CTLA4 inhibits glucose uptake and metabolism, maintaining the metabolic profile of non-activated T cells.^28^ PD-1 ligation also impairs glucose metabolism while additionally promoting fatty acid oxidation (FAO) of endogenous lipids both in the setting of primary activation and chronic antigen stimulation.^28,29^ Importantly, PD-1 signaling in chronically stimulated T cells induces a metabolically irreversible state not rescued by PD-1 blockade, which instead promotes reactive oxygen species (ROS) and cell death.^29^

#### Supporting pro-growth metabolism in T cells with mTOR and Myc

Downstream of co-stimulation and PI3K-AKT, the mammalian target of Rapamycin (mTOR) kinase pathway integrates multiple signals and regulates anabolic metabolic reprogramming in T cells exiting quiescence. mTOR complex 1 (mTORC1) is required for cell cycle entry and coordination of early metabolic changes that occur upon T cell activation. T cells deficient in Raptor, an essential component of mTORC1, fail to upregulate the expression of Glut1 and other glycolytic enzymes when activated.^30–33^ Raptor-deficient T cells also exhibit defects in de novo lipid synthesis and oxidative phosphorylation, suggesting the mTOR pathway is a global regulator of T cell metabolic programs.^34^ Beyond activation, mTORC regulates T cell metabolism in differentiated effector T cells. Raptor deficiency leads to impaired lipid biosynthesis and mitochondrial respiration in Tregs and B follicular helper T (Tfh) cells, although these results are model dependent.^33–36^ Regulation of mTOR signaling during T cell activation and differentiation is regulated by asymmetric cell division, which may explain these discrepancies.^37,38^ Memory T cells also require mTOR signaling to sustain effector metabolic programs. CD8 memory T cells deficient in Rictor, an essential component of mTOR complex 2 (mTORC2), are unable to sustain glycolysis upon reactivation, although initiation of glycolysis is Rapamycin insensitive.^2^

The mTOR pathway functions primarily through post-transcriptional regulation of key metabolic transcription factors, although effector transcription factors such as Tbet can be regulated directly by mTORC1 as well.^39^ Raptor-deficient T cells have diminished protein levels of two primary regulators of lipid synthesis, Sterol Regulatory Element Binding Proteins 1 and 2 (SREBP1 and SREBP2).^40^ Raptor knockout T cells also have reduced mRNA expression of genes that encode enzymes in the glycolysis as well as fatty acid and sterol biosynthesis pathways.^41^ mTORC1-mediated signaling is also required for proteomic remodeling of pathways including one-carbon metabolism, FAO, and the electron transport chain (ETC) that occurs early in activating T cells.^42^ mTOR also modulates T cell metabolism through control of hypoxia inducible factor 1 alpha (HIF1a) in some contexts. Loss of HIF1α in Th17 and CD8 T cells, but not other subsets or activation states leads to decreased expression of Glut1 and other key metabolic genes.^30,31,43,44^ Signaling through mTOR also regulates the key metabolic transcription factor c-Myc. Raptor deficiency results in loss of c-Myc protein without changing transcript levels, similar to the effect on SREBP genes, in early activated T cells.^41^

Myc mRNA and c-Myc protein levels in activated T cells are also directly regulated by TCR signaling and are sustained by IL-2-mediated signaling.^45–48^ Stabilization of c-Myc is essential for the global metabolic reorganization that occurs early in activating T cells. c-Myc deficiency results in a drastic reduction in T cell proliferative capacity and cell growth.^43,47^ These defects in c-Myc-deficient T cells are in part due to insufficient amino acid, lipid, and nucleotide precursor accumulation. c-Myc knockout T cells are also unable to increase glucose uptake, glycolytic flux, and the expression of enzymes required for glucose metabolism. Moreover, c-Myc loss impairs polyamine synthesis, due to both upstream defects in glutaminolysis and decreased expression of polyamine biosynthesis genes.^43^ Network analysis of proteomic alterations in c-Myc-deficient T cells shortly after activation also demonstrates a role for c-Myc in controlling mitochondrial ribosome biogenesis.^42^ These data together demonstrate the role of c-Myc as a master regulator of metabolic programming in activating T cells.

## Bottom-up metabolic signaling

As reviewed in the previous sections, primary activating signals from the TCR and co-stimulatory receptors drive many of the metabolic adaptations during T cell responses. This “top-down” control of metabolic protein expression and function through signal transduction and gene transcription is essential for establishing a framework through which biochemicals can be produced and consumed. However, in addition to extracellular signals, metabolites regulate these same signal transduction networks. This layer of regulation by metabolites represents “bottom-up” metabolic signaling whereby metabolites directly modify the activity of signaling effectors and gene transcription.

### Metabolic regulation of signaling effectors

Metabolites act as key regulators of signaling effector molecules, either through mechanisms that directly sense metabolite concentration or by providing the substrates required for functional protein modifications. In this section, we review the mechanisms of bottom-up metabolic signaling that regulate T cell activation and function described in the current literature.

#### AMPK: balancing energy homeostasis and T cell activation

One of the most well appreciated mechanisms of bottom-up metabolic signaling is the AMP-activated protein kinase (AMPK) signaling network. Cells must constantly regulate energy stores, primarily in the form of ATP, to coordinate energy-producing and energy consuming processes. AMPK binds to and is activated by adenine nucleotides, detecting when cellular energy stores are low by sensing the relative concentration of ATP to its low-energy enzymatic products AMP and ADP. A drop in cellular energy promotes AMPK’s kinase activity. Upon activation, AMPK phosphorylates components of cellular energetic pathways, including glycolysis, mitochondrial metabolism, and lipid metabolism.^49^ AMPK also activates the TSC complex, which inhibits mTORC1.^50,51^ Together, these actions promote catabolism to restore cellular ATP levels (Fig. 1).

AMPK is a critical regulator of T cell metabolism and function. Highly glycolytic activated T cells are unable to maintain ATP levels in low glucose environments. In this setting, the disruption of cellular energetics engages AMPK, which in turn inhibits mTOR activity, mRNA translation, and T cell proliferation. Loss of AMPKα1, the catalytic component of AMPK, is sufficient to restore mTOR signaling and T cell cytokine production, but not proliferation, when cells are stimulated in limiting glucose conditions. Although loss of AMPK can restore some functions in glucose-restricted T cells, the cells do not initiate metabolic adaptations necessary to recover ATP levels. In vivo, AMPKα1-deficient T cells have decreased mitochondrial respiration, flux of glutamine into the mitochondria, and ATP:AMP ratios, and therefore fail to proliferate and function effectively.^52^ These data illustrate how metabolic sensing, rather than a depletion of metabolic resources per se, can act upstream of T cell functional programming.

AMPK has been proposed to play an important role in sensing other metabolic changes associated with T cell activation, beyond energetics. T cell activation results in an increase in ROS. When T cell ROS levels are reduced through the use of scavengers, T cells display sustained AMPK signaling after activation. Consistent with this, ROS scavenger treatment results in impaired mTOR signaling throughout activation. Moreover, T cells treated this way exhibit diminished glucose uptake, glycolysis, and decreased proliferative capacity, in keeping with enhanced AMPK activity.^53^

#### Metabolic sensing through mTOR

The previously described mTOR pathway is another nutrient responsive pathway critical for regulating T cell responses. Apart from its regulation by PI3K-AKT and indirect sensing of changes in metabolism through AMPK, mTOR signaling is directly regulated through multiple metabolic processes (Fig. 1). Amino acids act as potent activators of mTORC1 signaling through multiple mechanisms. One well characterized mechanism is through amino acid binding to Rag GTPases, which bind to mTORC1 and localize the complex to Rheb-containing lysosomal compartments.^54,55^ Leucine further promotes Rag GTPase-mTORC1 interactions by relieving Sestrin inhibition of the pathway. Sestrins impair mTORC1 activity through their binding and negative regulation of GATOR2. GATOR2 indirectly promotes mTORC1 signaling by inhibiting GATOR1, which in turn is a negative regulator of Rag GTPases. When leucine binds Sestrin2, it relieves GATOR2 inhibition and potentiates mTORC1 activity.^56,57^

Other metabolic sensors can influence mTORC1 signaling through their inhibition of GATOR2. The CASTOR1/2 complex antagonizes mTORC1 signaling by negatively regulating GATOR2 when cellular arginine is limiting. High concentrations of cellular arginine relieve CASTOR1-mediated GATOR2 inhibition and thus promote mTORC1 activity.^58,59^ Methionine also regulates mTORC1 though the activity of GATOR family proteins. S-adenosylmethionine (SAM), a methionine metabolite, binds the recently identified SAMTOR protein, disturbing its interaction with GATOR1 and relieving mTORC1 inhibition.^60^ Amino acids activate mTORC1 through Rag GTPase-independent mechanisms as well. For example, in Rag GTPase-deficient cells, high glutamine concentrations activate mTORC1 through lysosomal translocalization via a mechanism dependent on protein ADP-ribosylation factor 1 (ARF1).^61^

Modulation of mTOR pathway activity by amino acids is critical for T cell activation. As has been documented in other cell types, amino acid deprivation results in potent inhibition of mTORC1 in activated T cells.^62^ Upon activation, T cells rapidly increase glutamine uptake through the glutamine transporter, ASCT2/Slc1a5, and require extracellular glutamine to proliferate.^43,63–65^ Evidencing the important role glutamine plays as a signaling molecule, T cells fail to properly engage mTORC1 signaling when they lack Slc1a5 or are cultured in glutamine-free media.^64,66^ Furthermore, glutamine supplementation is sufficient to partially restore signaling in amino acid-depleted media, and culturing T cells in high levels of glutamine can overcome the impact of Slc1a5 deficiency on mTORC1 activity.^64,66^

In addition to directly interacting with the mTOR pathway, glutamine has also been shown to indirectly regulate mTORC1 through glutaminase GLS1 and glutaminolysis in a Th cell program-specific manner. In the absence of GLS1 activity, Th1 cells display enhanced mTORC1 signaling, whereas Th17 cells show impaired activity.^65^ This differential effect of GLS1 on mTORC1 results from glutaminolysis promoting the Th1-specific expression of PI3KIP, a PI3K-mTOR signaling inhibitor. Formally demonstrating this mechanism, sgRNA targeting of PI3KIP in Th1 cells phenocopies GLS1 inhibition, enhancing mTORC1, while stimulating PI3KIP with cross-linking antibody yields diminished mTORC1 activity.^65^

While glutamine is an important regulator of mTORC1, T cells are particularly sensitive to leucine availability. Unlike glutamine, leucine is sufficient to restore mTOR activity in the absence of other exogenous amino acids. Selectively depleting leucine or blocking the neutral amino acid transporter that transports leucine, Slc7a5, impairs mTORC1 activity equivalently to pan-amino acid deprivation.^66^ Consistent with leucine playing a privileged role in regulating the mTOR pathway in activating T cells, impairing leucine processing through the branched chain amino acid catabolism pathway promotes mTOR activity. Upon T cell activation, both the transcript and enzymatic activity of the cytosolic isoform of branched chain aminotransferase (BCATc) are rapidly induced, leading to the production of the ketoacid α-ketoisocaproate. BCATc-deficient T cells have elevated mTORC1 activity and increased rates of glycolysis.^67^

Amino acid regulation of mTOR activity is not restricted to effector T cells, as has been recently highlighted by Chi et al.^68^ Tregs also rely on amino acid-induced mTORC1 activation. Like inflammatory effector T cells, Tregs display greater mTORC1 activity than their naïve counterparts. Tregs have increased transcript levels of the amino acid sensors Sestrin1, CASTOR1, CASTOR2 as well as components of the GATOR1 and GATOR2 complexes. Similar to their mechanism of action in other cell types, these amino acid sensors and the downstream effector GATOR complexes interact with RagA in activated Tregs, suggestive of functional activity. In vivo, mTOR activity is essential for Treg suppressive capacity. Selective deletion of RagA and RagB in FoxP3-expressing cells leads to spontaneous T cell activation, systemic inflammation, and early lethality.^68^ These data demonstrate the importance of amino acid sensing to homeostatic Treg function.

Beyond amino acid levels, mTOR signaling in T cells is also sensitive to changes in cellular ROS. T cell activation induces rapid production of ROS, as early as 15 min after activation.^69^ To cope with this burst in superoxide production, activated T cells engage in increased synthesis of the antioxidant glutathione (GSH). Both CD4 and CD8 T cells display elevated GSH levels within the 1st day of activation. This is accompanied by increased transcription of the catalytic subunit of glutamate cysteine ligase (GCLC). Accordingly, conditional deletion of GCLC in T cells results in a loss of activation-induced GSH synthesis and elevated ROS. Though GCLC-deficient T cells express markers of early activation, like CD44 and CD69, at normal levels, and show intact ERK-MAPK signaling, they fail to properly activate the mTOR pathway or to stabilize Myc expression. Consistent with this, loss of GCLC results in decreased glycolysis, proliferation, and cell size. Supplementation with either GSH or the ROS scavenger N-acetyl-cysteine is sufficient to restore mTOR and Myc in activated T cells and reverse the metabolic and cellular defects resulting from GCLC deficiency, illustrating the role of ROS sensing in the reprogramming of these networks.^70^

#### Lipids as metabolic mediators of T cell signaling

Working upstream and synergistically with mTOR signaling, many of the key initiating events of T cell activation are metabolic signaling reactions of membrane phospholipids. T cell receptor engagement leads to activation of phospholipase C (PLC), which hydrolyzes the membrane phospholipid phosphatidylinositol 4,5-biphosphate (PIP2) to generate IP3 and diacylglycerol (DAG).^7,71,72^ The lipid signaling mediators IP3 and DAG activate store-operated calcium signaling and protein kinase C, respectively.^73^ Co-stimulation and IL-2 signaling also promote alterations in membrane phospholipid content through PI3K recruitment to the cell membrane where it converts PIP2 to phosphatidylinositol 3,4,5-trisphosphate (PIP3), which serves as a docking site for PDK1 and its target AKT.^7^ Conversion of PIP2 to PIP3 by PI3K is antagonized by PTEN, a critical negative regulator of T cell homeostasis. Haploinsufficiency of PTEN results in a lethal T cell-mediated autoimmune disorder characterized by spontaneous T cell activation and reduced Fas-mediated activation-induced cell death.^74^ Conditional deletion of PTEN using the T cell-specific CD4-Cre in mice leads to an autoimmune phenotype characterized by spontaneous activation and differentiation of effector T cells.^75,76^ PTEN activity also regulates Treg function. PTEN-deficient Tregs rapidly proliferate in response to IL-2 stimulation alone, with restoration of PTEN expression sufficient to reverse this phenotype.^77^ More recently, Zou et al.^78^ identified the lipid kinase acylglycerol kinase (AGK) as a novel regulator of the PI3K-PTEN axis in CD8 T cells. AGK phosphorylates monoacyl glycerol and DAG to generate lysophosphatidic acid and phosphatidic acid, respectively. Upon TCR and CD28 stimulation, PTEN is recruited to the plasma membrane and interacts directly with and is phosphorylated and inactivated by AGK. In AGK-deficient CD8 T cells, PTEN remains in an active, unphosphorylated state leading to impaired AKT and mTOR signaling. AGK-deficient CD8 T cells are less proliferative, functional, and capable of controlling tumor growth in vivo than wild-type cells. Notably, this mechanism is specific to CD8 T cells, as CD4 T cells are not dependent on AGK for PTEN inactivation.

Beyond the role phospholipids play in signaling downstream of receptor activation, metabolic pathways play an important role in regulating the initiating T cell activation events. IP3 produced by PLC potently stimulates calcium efflux from the endoplasmic reticulum (ER) by engaging IP3 receptors, the initiating step of store-operated calcium entry. The sarco/ER Ca^2+^-ATPase (SERCA) channels mediate calcium uptake into the ER to restore the cytoplasm-ER calcium gradient and terminate calcium flux-mediated signaling. This metabolite-mediated process is further regulated by the glycolytic intermediate phosphoenolpyruvate (PEP). PEP inhibits SERCA channels leading to potentiated calcium signaling, integrating glycolytic activity and proximal TCR signaling.^79^ T cells activated in low glucose environments flux less calcium while exhibiting normal PLC, ERK-MAPK, and PI3K-AKT activity, indicating a mechanism independent of IP3. Inhibiting SERCA channels with thapsigargin or knockdown of the enzyme responsible for PEP production, enolase 1 (Eno1), is sufficient to restore calcium signaling and translocation of the Ca^2+^-activated transcription factor NFAT1 to the nucleus in T cells with impaired glycolysis. In vivo, enhancing PEP production through overexpression of PCK1, an enzyme that produced PEP from oxaloacetate, is sufficient to enhance CD4 and CD8 T cell function in murine tumor models, suggesting that glycolytic regulation of signaling is a mechanism by which glucose-replete tumors evade immune responses.^79^

#### The cholesterol biosynthesis pathway and T cell signaling

T cell signaling can be modulated by several other lipid species, particularly those within the cholesterol biosynthesis pathway. T cells rapidly increase their cholesterol stores and the expression of cholesterol biosynthesis genes upon activation.^40,80–82^ Though a major role for this increase in cholesterol biosynthesis is to support membrane production for proliferation, the pathway is also appreciated to control T cell signaling at multiple levels.

Engagement of the TCR and co-stimulation results in a marked restructuring of the plasma membrane.^83–85^ As part of this process, lipid species condense at the site of the TCR and display an altered lipid composition.^86–90^ Depletion of CD8 T cell plasma membrane cholesterol impairs TCR receptor clustering and signaling; conversely enhancing plasma membrane cholesterol localization by impairing the cholesterol esterase Acat1 results in increased TCR clustering and signaling.^91^ These detergent-resistant clusters of cholesterol and phospholipids, known as “lipid rafts”, are rich in kinases and signaling scaffolds critical for the initiating events of T cell activation.^86,88,92–94^ The accumulation of lipid rafts at the site of the TCR during antigen presentation has been proposed to concentrate these signaling effectors and permit T cell activation.^88,92,95^ Although membrane cholesterol deposition can contribute to T cell signaling, signaling microdomains can form in the absence of lipid rafts through protein–protein interactions and disruption of lipid raft formation does not impair microdomain clustering.^96,97^

In addition to controlling plasma membrane structure, the cholesterol biosynthesis pathway has the ability to impact the localization of signaling effectors through prenylation. Farnesyl pyrophosphate (FPP) is a common precursor of both cholesterol and isoprenoids. Both FPP and the downstream product geranylgeranyl pyrophosphate (GGPP) can modify and activate signaling proteins by localizing them at the plasma membrane.^98^ In T cells, prenylation regulates ERK signaling downstream of the TCR through prenylation of RhoA and farnesylation of Ras.^99^ Depletion of isoprenoid stores or use of prenylation inhibitors impairs T cell proliferation and Th1 differentiation.^99,100^ Impairment of prenylation has also been shown to promote Treg differentiation at the expense of Th17 cells, downstream of TGF-β signaling.^101,102^

Intracellular sensing of cholesterol species plays an important role in T cell activation and differentiation. Cellular sterol stores are predominantly sensed and controlled by the SREBP and LXR families of transcription factors.^103,104^ Suppression of LXR signaling and enhancement of SREBP signaling are critical steps during T cell quiescence exit to promote proliferation and growth.^40,82^ After T cells activate, cholesterol sensing by LXR and SREBP also plays an important role in differentiation. LXR signaling impairs Th17 differentiation, with Srebp-1 expression being responsible downstream of LXR.^105^ Consistent with this, LXR agonists suppress IL-23R expression and diminish EAE severity.^106^ In contrast, LXR activation supports Treg differentiation at the expense of the Th1 and Th17 cell programs.^107^ Sterol sensing can regulate Th17 differentiation even more directly as sterol binds to RORγt and drives its activity to serve as a transcription factor, while treatment with statins impairs RORγt expression.^108,109^

#### Modulation of T cell activation by glucose

In addition to metabolic sensing by signaling effectors and transcription factors during T cell activation, metabolic enzymes themselves have been found to act as sensors that toggle T cell function. Glycolytic enzymes act as direct regulators of T cell function through mechanisms independent of catalytic activity. Glyceraldehyde 3-phosphate dehydrogenase (GAPDH) catalyzes the sixth step of glycolysis and also acts as an mRNA-binding protein.^110,111^ In multiple cell types, enzymatically inactive GAPDH binds mRNA and inhibits translation.^112–116^ This inhibition is relieved when glycolytic flux is elevated. In T cells, GAPDH binds the key effector molecule transcript Ifng to control translation, linking glycolytic activity directly to effector function.^117^ Mechanistically, GAPDH associates with an AU-rich region of the 3′ untranslated region (UTR) of the Ifng mRNA, limiting protein translation when GAPDH is insufficiently engaged in catalytic activity. Further studies are required to define the complete set of RNAs bound by GAPDH in various T cell stimulation conditions to determine how else glycolytic activity influences the proteome.

Glucose and glucose-derived metabolites regulate T cell activation and signaling through modulation of receptors. The majority of plasma membrane receptors and transporters are thought to be N-glycosylated within the ER.^118^ N-glycosylated proteins are further modified in the Golgi apparatus by the MGAT family of N-acetylglucosaminyltransferases that require UDP-N-acetylglucosamine (UDP-GlcNAc). UDP-GlcNAc is synthesized by the hexosamine pathway using the substrates and glycolysis intermediate fructose-6-phosphate as well as glutamine, Acetyl-CoA (Ac-CoA), and UTP. This pathway integrates central carbon, amino acid, and nucleotide metabolism.^119^ Therefore, glycosylation acts as a mechanism by which metabolic information is integrated into protein function.

T cell activation induces the expression of MGAT protein, increases intracellular UDP-GlcNAc biosynthesis, and results in elevated protein N-glycosylation.^120–125^ Several regulators of T cell activation are actively modified by N-glycosylation, including the TCR, the CD4 and CD8 co-receptors, inhibitory receptors such as CTLA4, and cytokine receptors including the TGF-β receptors.^124,126^ In T cells, MGAT5 has been found to be the dominant regulator of glycosylation. Early studies using inhibitors of N-glycosylation demonstrated that depletion of MGAT5-modified glycans enhances T cell proliferation.^127^ Corroborating these findings, MGAT5-deficient mice develop spontaneous autoimmune disease due to diminished TCR signaling thresholds due to increased CTLA4 endocytosis and a loss of N-acetylgalactosamine-galectin lattices that restrict the TCR to sites of antigen presentation.^120,123^ This is an established mechanism by which galectins impair T cell activation and induce apoptosis when present at high levels.^128,129^ Extracellular lattices partition T cell signaling complexes by counteracting cytoskeleton to inhibit naïve T cell activation. When antigen is absent, galectin maintains CD45 in TCR microdomains and impairs recruitment of WASP and suppressive signaling.^130^

N-glycosylation also plays a critical role in thymocyte selection. MGAT5-mediated branching is a critical factor of TCR-MHC affinity. First, N-glycosylation negatively regulates high-affinity TCR activity, allowing cells with these receptors to survive negative selection. Secondly, N-glycosylation enhances surface expression of CD4 and CD8 co-receptor, allowing cells expressing low-affinity TCRs to survive positive selection.^126^ MGAT5 also modulates mature Th cell differentiation in the periphery. MGAT5-deficiency impairs IL-4 and enhances IFNγ production in Th2-skewing cell culture conditions, suggesting that N-glycosylation restricts aspects of Th1 cell differentiation.^122^ Consistent with these data, T cells from animals fed GlcNAc or in vitro T cells treated with the same metabolite have increased N-glycosylation and are defective in Th1 and Th17 cell differentiation.^123,131^ Highlighting the importance of N-glycosylation in Th cell differentiation, the Th17 metabolic program actively antagonizes the hexosamine pathway. Inflammatory T cells readily engage glycolysis and Th17 cells favor glutaminolysis. Both of these metabolic pathways deprive carbon and nitrogen from UDP-GlcNAc biosynthesis and enhance Th17 cell differentiation. Anti-inflammatory Tregs are more dependent on oxidative phosphorylation, which increases flux into the hexosamine pathway and N-glycosylation. This results in increased CD25 glycosylation, stabilizing its expression in the cell surface to promote Treg maintenance.^132^

In addition to N-glycosylation, O-glycosylation has also been found to regulate T cell biology. UDP-GlcNAc can also be metabolized by O-GlcNAc transferase (OGT) to reversibly add a O-GlcNAc modification to serine and threonine residues of proteins.^133^ In contrast to the role of N-glycosylation on cell membrane proteins, O-GlcNAcylation acts on intracellular proteins, where it can limit accessibility of serines and threonines to phosphorylation and modulate kinase signaling.^134–136^ Activation of T cells results in a rapid increase in protein O-GlcNAcylation.^137–139^ In particular, T cell O-GlcNAcylation impacts nuclear proteins, including positively regulating the activity of NFAT and NF-κB subunits.^137,140^ Beyond T cell activation, OGT activity is also critical during thymic development as well as malignant transformation of the T lineage.^139^ In this manner, glucose and glutamine uptakes work in concert to modulate T cell signaling from receptors on the cell surface to transcriptional regulators within the nucleus, through N- and O-glycosylation.

### Transcriptional regulation

#### Mitochondria-cytosolic crosstalk and histone acetylation in differentiating T cells

**Fig. 2:**
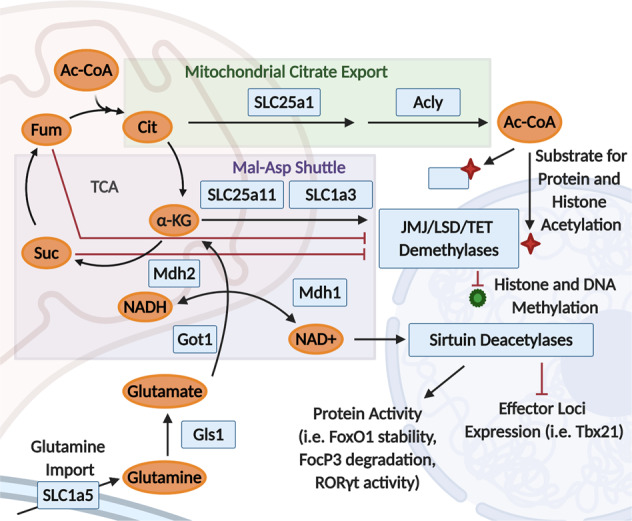
Metabolic regulation of transcription. Crosstalk between metabolites generated by citrate cycle (TCA) reactions in the mitochondria and effectors in nucleus/cytosol regulate protein and histone post-translational modifications. Mitochondrial citrate export through the citrate transporter SLC25a1 is a required source of extra-mitochondrial Ac-CoA for protein and histone acetylation. The malate-aspartate shuttle regulates concentrations of α-ketoglutarate (α-KG), succinate (Suc), fumarate (Fum), and FAD, which regulate the activity of the JMJ, LSD, and TET family demethylases. This shuttle also regulates the redox status of the cell, controlling the activity of NAD-activated sirtuin deacetylases.

Cellular metabolism supports cell program-specific epigenetic remodeling in addition to providing substrates for biomass generation and protein modification.^141,142^ Many metabolic adaptations that result from T cell activation are interconnected with epigenetics. One of the primary pathways that connect metabolism to epigenetic regulation is the export of mitochondrial citrate to the cytosol via the mitochondrial citrate carrier (Slc25a1). Citrate is an abundant mitochondrial metabolite produced in an early TCA cycle reaction by citrate synthase from mitochondrial Ac-CoA and oxaloacetate (OAA). Cytosolic metabolism of citrate by ATP citrate lyase (Acly) regenerates Ac-CoA in this compartment, providing the main pool of this substrate required for histone acetylation^143^ (Fig. 2).

Activated T cells rely on citrate export from the mitochondria to support extensive epigenetic remodeling required for differentiation. Lactate dehydrogenase A (LDHA)-deficient CD4 T cells have impaired glycolytic function and produce less IFNγ, indicating a functional defect.^144^ To compensate for decreased glycolytic function, these cells increase metabolic flux through the TCA cycle, decreasing the pool of citrate for mitochondrial export and cytosolic Ac-CoA production. Decreased cytosolic Ac-CoA concentrations directly modulated T cell effector functions, leading to decreased histone acetylation at the *Ifng* promoter and CNS-22 enhancer, but not at other gene bodies such as *Cd3e*. How gene locus specificity is accomplished and whether Ac-CoA production occurs in other cellular compartments such as the nucleus is an area of open investigation. Supporting a role for glycolysis in promoting T cell reprogramming via citrate export, metabolic inputs that enhance glycolysis promote Acly and acetylation-dependent changes in T cell function. Supplementation of Th17-cultured T cells with the short-chain fatty acids pentanoate or acetate enhances IL-10 production. These supplementation conditions enhance glycolysis, Ac-CoA production, and histone acetylation at the *IL-10* promoter by a 2DG and Acly inhibitor-sensitive mechanism.^145^ More recently, direct genetic evidence demonstrates the critical role of citrate export in T cell epigenetic reprogramming. Using a CRISPR-Cas9-based approach in vitro, deletion of either *Slc25a1* or *Acly* in activated CD4 T cells resulted in a loss of total cellular H3K9 acetylation and a decrease in IFNγ production.^146^ The results of this study suggest that widespread changes to the histone acetylome occur as a result of Ac-CoA deprivation, and further studies are required to determine the mechanisms that regulate locus-specific modifications. These studies together highlight the importance of metabolic exchange between the mitochondria and the cytosol to pattern T cell differentiation.

The regulated transport of metabolites between metabolically compartmentalized organelles is required to maintain substrate availability for various processes (Fig. 2). Beyond the example of citrate export presented above, the malate-aspartate shuttle is another transport system that regulates T cell function. Together, these shuttling systems effectively involve the first and last steps of the TCA cycle to run forward in the mitochondria and then reverse in the cytosol, with the net effect being the movement of electrons into the mitochondria (in the form of NADH) and carbon into the cytosol (in the form of Ac-CoA). The malate-aspartate shuttle consists of a cycle in which cytosolic OAA and NADH are converted into malate and NAD^+^ by malate dehydrogenase 1 (Mdh1), and then mitochondrial malate is oxidized to OAA by malate dehydrogenase 2 (Mdh2), generating mitochondrial NADH. Cytosolic and mitochondrial pools of malate and OAA are connected by two transporters — the malate-2OG transporter (Slc25a11) and the glutamate-aspartate transporter (Slc1a3) — and by the interconversion of aspartate and glutamate into OAA and 2OG by the cytosolic and mitochondrial isoforms of the glutamate-oxaloacetate transaminase, Got1 and Got2, respectively. OAA can also be used to generate citrate in mitochondria by citrate synthase (Cs), which in turn may be transported to the cytosol using Slc25a1 and cleaved by Acly back into OAA and Ac-CoA.

The malate-aspartate shuttle is required for CD4 T cell activation and differentiation. CRISPR-Cas9 targeting of any of the enzymes or transporters of the malate-aspartate shuttle results in impaired IFNγ cytokine production in activated Th1 cells.^146^ Loss of malate-aspartate shuttle activity also leads to reduction in H3K9 acetylation, suggesting a connection between this pathway and the citrate export pathway previously described. Formally demonstrating that the activity of the shuttle network, rather than the TCA cycle, regulated CD4 T cell biology, targeting either shuttle or the cytosolic isoform of Mdh1 was sufficient to impair mitochondrial respiration. In addition to the impact of the malate-aspartate shuttle on histone acetylation, the shuttling network was also found to be essential for T cell proliferation through its control of respiration.^146^ Similar to what has been found in cancer cells,^147,148^ Complex-I activity regenerates NAD^+^ that allows cytosolic aspartate to be produced by GOT1, which is necessary for nucleotide biosynthesis. Indeed, impairing either Complex-I with the inhibitor rotenone or targeting the malate-aspartate shuttle results in a block in the synthesis of aspartate and its downstream nucleotide precursor, N-carbamoyl-l-aspartate. In this manner, CD4 T cell division is impaired when cells are treated with rotenone, but can be restored simply by supplementing with exogenous aspartate.^146^ It is therefore likely that the malate-aspartate shuttle contributes to histone remodeling both by interacting with citrate export as well as by supporting cell proliferation.

#### Metabolic regulation of deacetylation by sirtuins

The NADH/NAD^+^ ratio also directly impacts histone and protein acetylation by controlling the activity of NAD^+^-dependent sirtuin deacetylases^149,150^ (Fig. 2). Sirtuin 1 (SIRT1)-deficient T cells spontaneously activate, displaying a breakdown in tolerance.^151^ These cells are also resistant to in vitro anergy induction.^152^ However, the T cell-driven pathological phenotype in SIRT1 knockout mice is likely contributed to by extrinsic factors, as T cell conditional knockout mice do not develop disease or have dysfunctional effector T cells.^153^ Mechanistically, SIRT1 deacetylates the *Tbx21* locus in CD8 T cells, and is suppressed by AP1 family members BATF and c-Jun to promote Tbet expression in CD8 T cells.^154^ Although the sirtuin family proteins are reported to regulate histone acetylation in CD4 T cells, this is a largely unexplored aspect of the metabolic-epigenetic axis warranting further study.^155^

Sirtuin family members are also key regulators of post-translational modifications to non-histone proteins. Several studies have documented the role of sirtuin proteins in regulating acetylation of key T cell-associated transcription factors. SIRT1 is critical for maintaining FoxO1 protein stability and sustaining oxidative phosphorylation in resting CD8 T cells.^156^ In the context of T cell anergy, SIRT1 negatively regulates c-Jun to suppress T cell activation.^151^ This axis specifically is the target of the HIV Tat protein, which directly interacts with SIRT1 to suppress deacetylation of the p65 subunit of NF-κB to promote T cell proliferation.^157^

Perhaps the best characterized role for SIRT1 in T cells is in Treg biology. Acetylation blocks ubiquitination and proteasomal degradation of FoxP3. Active SIRT1 deacetylates FoxP3, allowing for its subsequent degradation.^153,158,159^ Impairing or knocking out SIRT1 in Tregs leads to increased suppressor function and promotes the acceptance of allografts in murine transplant models.^153,158^ Other sirtuin family members regulate Treg cell biology. Similar to SIRT1, SIRT4 regulates FoxP3 stability and Treg cell function.^160^ More generally, SIRT1 regulate the balance of Th17-Treg differentiation. T cell-specific deletion of SIRT1 and SIRT1-specific inhibitors suppress the Th17 program and are protective in mouse models of experimental autoimmune encephalomyelitis (EAE). Mechanistically, active SIRT1 deacetylates the key Th17 cell transcription factor RORγt, enhancing its activity.^161^

#### Mitochondrial control of histone methylation

In addition to regulating histone and protein acetylation, alterations in metabolic flux control the availability of substrates that regulate protein methylation (Fig. 2). Intermediates of the TCA cycle, 2-oxoglutarate (2OG) and FAD, are obligate cofactors of the two main classes of histone demethylases, 2OG-dependent dioxygenases (JMJ family) and FAD-dependent amine oxidases (LSD family). Additionally, metabolites of the TCA cycle negatively regulate demethylase activity. For example, succinate and fumarate are potent allosteric inhibitors of the 2OG-dependent dioxygenases. Therefore, the production and consumption of these metabolites, and their transport from the mitochondria to the cytosol dictates histone dynamics in the nucleus.

2OG-dependent demethylation is a critical regulatory step in T cell activation and differentiation. Mitochondrial 2OG is generated by the TCA cycle or by glutaminolysis and transamination of glutamate. In activated effector T cells, glutamine catabolism and transamination is the major source of 2OG synthesis.^43,65,162^ Accordingly, many of the transcriptional defects that occur as a result of glutamine deprivation or GLS inhibition can be rescued with 2OG supplementation.^65,163^ These data link the TCA cycle and glutamine metabolism to epigenetic remodeling in T cells. Highlighting the importance of 2OG to T cell transcriptional reprogramming, 2OG regulates roughly a third of all IL-2-induced genes. This regulation occurs through JMJD3 and TET2-mediated removal of H3K27 trimethylation (H3K27me3) and DNA methylation, respectively, at IL2-regulated loci. In this model, 2OG-dependent epigenetic modifications altered CTCF binding dynamics and chromatin structure.^163^ These results illustrate the dramatic role that metabolism can play in altering the genomic architecture of differentiating cells.

Several studies have demonstrated how T cell production and consumption of 2OG can have program-specific effects on epigenetic remodeling during T cell differentiation. Compared to Th1 and Treg cells, Th17 cells have higher rates of glutaminolysis and rely on this pathway for functional differentiation.^65^ Downstream of the glutamine-to-glutamate conversion catalyzed by GLS1, GOT1 acts as the dominant aminotransferase that generates 2OG from glutamate.^162^ Unlike Th1 cells that do not require GLS1 for effector cytokine production, GLS1-deficient Th17 cells produce less IL-17 than wild-type cells. Conversely, loss of GLS1 activity promotes Th1 cell cytokine production and Tbet expression.^65^ Further demonstrating the importance of glutaminolysis in Th17 cell differentiation, inhibition of GOT1 with aminooxyacetic acid (AOA) promoted the conversion of Th17 cells to FoxP3-expressing Tregs. shRNA targeting of GOT1 phenocopied this result, suggesting the activity of AOA is indeed through inhibition of glutaminolysis.^162^ Conversely, glutamine deprivation favors Treg differentiation and proliferation in both mouse and human T cells, even in the absence of TGFβ.^164,165^ These program-specific glutaminolysis inhibition phenotypes are mediated by 2OG-dependent methylation. Inhibition of GLS1 enhances H3K27me3 in Th17 cells, favoring differentiation of Th1 cells in a JMJD3-dependent mechanism. Strikingly, these results are entirely dependent on the Th cell program being engaged. In contrast to cells stimulated in Th17-skewing conditions, cells treated with IL-12 and IFNγ exhibit enhanced chromatin accessibility and elevated transcription upon GLS1 inhibition.^65^ Further studies are required to determine the program-specific mechanisms that account for these differences in effector T cell subsets.

Glutaminolysis also regulates T cell differentiation by modulating DNA demethylation. ^13^C-labeled glutamine metabolic flux studies revealed that Th17 cells preferentially metabolize 2OG to 2-hydroxyglutarate (2HG), compared to Tregs. In cancer cells, 2HG is a competitive inhibitor of 2OG-dependent dioxygenases, including TET and JMJ family demethylases.^166–169^ 2HG also regulates CD8 T cell demethylation. Upon activation, CD8 T cells rapidly accumulate 2HG that promotes H3K27me3 and enhances H3K4me3 at promoter regions, with only minor effects on DNA demethylation.^170^ In contrast, 2HG is a critical regulator of DNA methylation in CD4 T cells. Upon treatment with 2HG, Treg cells convert to Th17 cells, even when maintained in Treg cell culture conditions. Moreover, 2OG supplementation partially rescues AOA treatment or knockdown of the enzymes responsible for 2HG production, IDH1 and IDH2. These phenomena are at least in part explained by the activity of 2HG as an inhibitor of TET enzymes. Treatment with 2HG promotes DNA methylation of the *FoxP3* promoter in both Th17 and Treg culture conditions.^162^

## Conclusions and future outlook

Our understanding of immune cell metabolism and the interconnectedness of signaling with metabolic networks has changed rapidly over the past decade. The traditional view of metabolism as a “house-keeping” program that sustains all cells in a uniform way has been shown as inadequate in explaining the biochemical diversity seen particularly in the immune system. As described above, many of these core metabolic processes can play vastly different roles within the same lineage of cells, depending on the functional program they adopt. This can even mean that some cell programs display a critical dependency on a pathway, while an alternate program is impeded by the same process. These discriminating metabolic features are both driven by the signaling pathways that dictate differentiation to sustain the biology of a given program and can act upstream of those same signaling networks to direct programming. Ultimately, cells must generate the biochemical landscape needed to sustain the transcriptome and proteome launched by differentiating cues. This is ensured by incorporating metabolic information as an input in signaling.

While this paradigm is now well established, there remain many outstanding questions. The field is just beginning to explore how metabolic interconnectivity of organelles regulates immune cell biology. Glutaminolysis, citrate export, and the malate-aspartate shuttle all connect the mitochondria to the cytosol and in turn to the nucleus. How other shuttling networks and the transport of metabolites in and out of the mitochondria pattern differentiation has only just begun to be explored. Moreover, we lack insight into how these changes mediate such profoundly specific alterations to gene expression when they affect seemingly fundamental and cell-wide processes (i.e., Ac-CoA availability). Zooming another level out, we know very little about how the flow of metabolites between interacting cells can mediate cell-to-cell communication or influence function. This is of particular interest within the immune system, whereby lymphocytes go from solitary circulating cells to fixed in intimate cell contact during antigen presentation and cell killing and the intercellular relationships between resident immune cells in organ systems. Indeed, at the level of organ systems, we know very little how responses to infection or other alterations in homeostasis in one system (e.g., the liver) change organismal homeostasis to alter immune responses. Illuminating how this higher order metabolic architecture is organized through the lens of metabolic signaling offers the opportunity for the development of a new wave of metabolic therapies for cellular and host pathology.
